# A Deep-Learning Model for Real-Time Red Palm Weevil Detection and Localization

**DOI:** 10.3390/jimaging8060170

**Published:** 2022-06-15

**Authors:** Majed Alsanea, Shabana Habib, Noreen Fayyaz Khan, Mohammed F. Alsharekh, Muhammad Islam, Sheroz Khan

**Affiliations:** 1 Computing Department, Arabeast Colleges, Riyadh 13544, Saudi Arabia; malsanea@arabeast.edu.sa; 2 Department of Information Technology, College of Computer, Qassim University, Buraydah 52571, Saudi Arabia; 3 Department of Computer Science, Islamia College University, Peshawar 25120, Pakistan; noreen.fayyaz@fu.edu.pk; 4 Department of Electrical Engineering, Unaizah College of Engineering, Qassim University, Unayzah 52571, Saudi Arabia; m.alsharekh@qu.edu.sa; 5 Department of Electrical Engineering, College of Engineering and Information Technology, Onaizah Colleges, Unayzah 56447, Saudi Arabia; m.islam@oc.edu.sa (M.I.); sheroz@oc.edu.sa (S.K.)

**Keywords:** red palm weevil, localization, classification technique, deep learning approach, region convolution neural network

## Abstract

**Background and motivation:** Over the last two decades, particularly in the Middle East, Red Palm Weevils (RPW, Rhynchophorus ferruginous) have proved to be the most destructive pest of palm trees across the globe. **Problem:** The RPW has caused considerable damage to various palm species. The early identification of the RPW is a challenging task for good date production since the identification will prevent palm trees from being affected by the RPW. This is one of the reasons why the use of advanced technology will help in the prevention of the spread of the RPW on palm trees. Many researchers have worked on finding an accurate technique for the identification, localization and classification of the RPW pest. This study aimed to develop a model that can use a deep-learning approach to identify and discriminate between the RPW and other insects living in palm tree habitats using a deep-learning technique. Researchers had not applied deep learning to the classification of red palm weevils previously. **Methods:** In this study, a region-based convolutional neural network (R-CNN) algorithm was used to detect the location of the RPW in an image by building bounding boxes around the image. A CNN algorithm was applied in order to extract the features to enclose with the bounding boxes—the selection target. In addition, these features were passed through the classification and regression layers to determine the presence of the RPW with a high degree of accuracy and to locate its coordinates. **Results:** As a result of the developed model, the RPW can be quickly detected with a high accuracy of 100% in infested palm trees at an early stage. In the Al-Qassim region, which has thousands of farms, the model sets the path for deploying an efficient, low-cost RPW detection and classification technology for palm trees.

## 1. Introduction

The red palm weevil has been demonstrated to be one of the most destructive insects of palm trees that attacks a variety of palm species (e.g., date palms, coconut palms and royal palms). In the early part of the 20th century, its presence was recognized as a medical condition in Southeast Asia. Furthermore, its presence has caused damage in the western and eastern parts of Asia as well as in northern Africa and Europe [1,2]. The RPW had spread by the end of the 20th century and was discovered in the western parts of Northern America by the end of the decade. The high spread rate is attributed primarily to human movement, by which young and adult date palm trees are moved from contaminated areas to areas without the RPW. This species of weevil has a life cycle that lasts from 45–139 days inside the trunk of a palm tree, where it feeds on the palm tissue. Because the RPW is inside the palm tree, it is protected and cannot be seen from the outside. Infected palm trees may remain infected for generations if food is available, but when the tree is hollow, the RPW usually leaves the palm tree to look for a new host. Throughout its life cycle, it has the forms of four different stages, which are egg, larva, pupa and adult.

As a result of the RPW ferruginous Olivier, date palm trees suffer considerable economic losses. The larvae invade the trunks of palm trees after hatching in low, wounded or sheltered areas of each tree, creating cavities and tunnels that weaken the tree’s structure by interfering with the communication of nutrients and water between the root system and crown. As soon as the signs of significant damage appear on the palm trees, secretive larvae normally appear. It is usually these unnoticeable larvae that get transported within a specific region or in between various agricultural regions. The early detection methods may therefore prove to be very effective in reducing the spread of these pests. In addition to providing the core component of nutrition for any social gathering, the date palm forms an integral part of the overall heritage of the Arabian Peninsula.

A recent invasion of the red palm weevil (RPW), which originated from Southeast Asia, is threatening this precious heritage [3]. The Saudi Arabian government has carried out a national campaign for the control of the RPW by destroying or containing infested plants, injecting and spraying them with biochemical and chemical treatments with a pesticide in heavily infested and newly infested areas, and using pheromone and kairomone traps to track and reduce RPW populations, but this campaign has been only partially successful in preventing the spread to unaffected areas. There is a need for new methods to help minimize the number of RPW populations. Recently, however, some countries developed methods to facilitate the early detection of an RPW epidemic before it spreads widely. These methods, if successful, will prove to be of great help to farmers in reducing or eliminating pests from their fields.

In order to manage and control RPWs, the current approaches include first detecting the presence of RPWs. These approaches have been modified to provide more accurate insect identification results in real time in place of a lack of manual identification methods for entomologists. The use of computer vision technology through pattern recognition has proven to be more productive when used to identify and classify insects [4]. Insects are composed of several parts, such as their antennae, tails, wings and so on. In an image processing process, these components are extracted for the purpose of using them in the identification of insects and their other important characteristics, such as their colors and shapes. There are several advantages to the new intelligent system, notably that it is particularly useful for lay people who do not have the professional knowledge to identify certain types of insects. Therefore, the automated system will reduce both the problem as well as the labor effort needed to increase the income of a farmer. The farmer will be encouraged to increase the yield of their date fruit if this is done.

In fact, many of the current RPW management strategies are based on manual applications of insecticides that may cause harm to the environment and the human body. There has not been much use of an automatic species identification for the red palm weevil (RPW) due to the complexities and high costs of such systems [5].

## 2. Related Work

Due to the serious problem of the red palm weevil infestation of date trees in Saudi Arabia, researchers from around the world have been actively involved in finding software and hardware approaches to successfully identify insects in date-tree plantations.

Cheong [6] used Integrated Pest Management (IPM) in a battle against the RPW insect. This is one of the most efficient methods for getting rid of this insect. In response to all the problems associated with the use of traditional labeling methods, Photographic Identification Methods (PIMs) were proposed as an alternative. The aim was to avoid pain, injury and stress to animals while at the same time allowing for individual identifications. Because of this, PIMs are some of the most popular techniques that are implemented, with many available software packages enabling the identification of different types of species individually. It is believed that these techniques require the fixed part of an organism that is common to all insects of the same species but has the organism’s own distinctive features [7]. 

Some species may not be suitable for PIMs, and the software will not recognize species that do not have natural patterns that reflect them. In these circumstances, traditional techniques are still the best option to be used. As an example, suitable photo-identifiable animal features include scars on fins, scales or color patterns [8]. In the past, PIMs have been used mainly on vertebrates, such as fish, amphibians, reptiles, birds and mammals. There have been only a few studies that have applied PIMs to invertebrates. 

In the past few years, several automated systems have been proposed to identifiably recognize different insects, such as the Automated Bee Identification System (ABIS) that was designed for identifying bees, as well as a proposed Digital Automated Identification System (DAISY) that was designed for identifying Ophioninae [9]. The Automated Insect Identification through Concatenated Histograms of Local Appearance (AI-ICHLA) system was developed and proposed as a method for the identification of stonefly larvae, and the Automated Species Identification and Web Access (SPIWA) system for the classification of spiders was developed and proposed as a method for the identification of the pecan weevil. 

Rach et al. [10] mentioned that this research focused on the identification of insets, such as butterflies and ladybugs. Using a color information system, the image of an insect is acquired and noise is suppressed using a color image-processing algorithm. The edge detection technique is applied to an RGB space after pre-processing by means of a top-cover filter with a specific threshold. In order to determine an observed edge line, a string symbol is analyzed. In order to improve the quality of the results, the image is filtered to a maximum and minimum. Yang et al. conducted a similar study in which the method of recognizing insects was based on pattern-recognition technologies. A pattern-recognition system can accurately be defined as the process of collecting raw data and taking action on the basis of the recognition of a pattern category. The authors explained that this process is divided into three stages: entering the data and information collected from the sample using a sensor; using a feature extraction mechanism that computes numeric or symbolic data from a sample; and using a classification scheme that identifies the group of samples. 

Image segmentation is defined as a method by which an image is divided into several parts, which are grouped together by using pieces that bear similar properties, such as density or texture, in order to produce an image without any overlaps. In previous studies, two different image-processing techniques were applied to identify and recognize the RPW based on images. The algorithm used in the present method makes use of the local features of an insect image as well as moment invariant values (Zernike moments). The processing time for RPWs and the other insects was found to be 0.47 s with 97% and 88% recognition rates, respectively. The same problem was solved with another method, whereby pixel information was sent to the ANN in binary form. It was estimated that training the said network would take 183.4 s, but a decision was made very quickly. According to the study, the best identification rates in terms of RPW and other insects were recorded to be 99% and 93%. 

The ANN proposed in this study has four layers and consists of a total of 24,771 neurons. The benefits of this method have been shown to be better results at a higher cost in terms of computational requirements [11]. One study obtained a framework for identifying the RPW based on a support vector machine (SVM) strategy and descriptors extracted from standard image preparation systems used in RPW identification. 

The development of a neural system based on parallel images (pixel information) using a framework was recently been published. This technique proved to be too computationally costly for practical field use. In particular, the test times for each picture were generally extremely long and the memory requirements for storing the binary pictures were too restrictive for practical field applications. As a result, various SVM-based pattern recognition techniques have been adopted for machine-vision applications, such as for face recognition problems, processed speech recognition and a simulated annealing algorithm for recognizing stored-grain pests. 

The pecan weevil was proposed to be identified by an identification system. Several imaging techniques have been proposed that rely on template matching to identify the pest [12]. An IoT-based smart palm-weevil monitoring system was developed based on using a web/mobile interface to detect the red palm weevil via sensors [1]. By applying 10 state-of- the-art data mining algorithms for classifications, tremendous work was done in 2021. It was estimated that these algorithms perform with an accuracy rate of 93% [13]. 

A deep-learning technique is one of the most important amongst the various classifier techniques that provide varying methods of identifying and classifying objects, including insects. Computer software tools are increasingly coming into use in the fields of agriculture, crop- and weed-detection differentiation and control. The faster R-CNN model was used to develop a Regional Convolutional 3D Network for object detection [14,15,16]. The use of the IoT-enabled environments was addressed by using information technology for realizing the implementation of smart cities [17]. The deployment of robot design, image acquisition and apple-detection quality evaluation with a more detailed description of apple-harvesting is given in [18]. Soil analysis and characteristics, the detection and classification of crop weed control and taste and odor detection are covered in detail in [19].

Researchers have not applied the deep-learning approach to the classification of red palm weevils. We used the faster R-CNN algorithm in order to detect the presence of the red palm weevil in this study. The purpose of using faster R-CNN was that it is an end-to-end single-stage model. It works on generating a region’s proposals, which saves time compared to traditional algorithms.

## 3. Proposed Model

**Figure 1:**
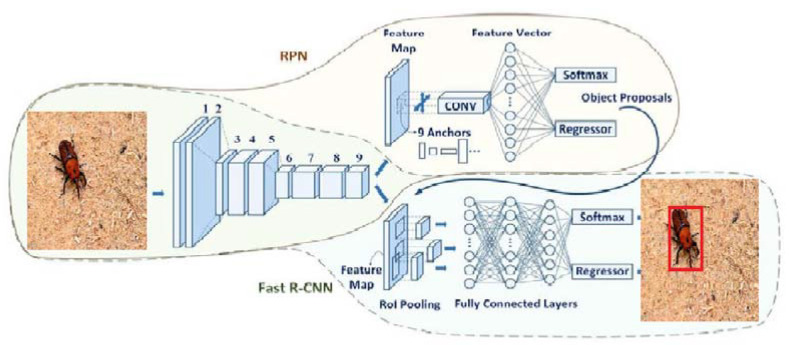
Proposed framework for the developed model.

In computer vision, object detection is a difficult and tedious task that involves recognizing the location of objects in an image and identifying the type of object that is detected. In reality, object detection is a very difficult problem to solve. There are two main steps in the process: (1) object localization, which involves identifying the positions of objects in an image, and (2) classification, which aims to identify the types of objects contained in an image. Many researchers have presented different methods for detecting objects in an image. In this study, we present methods to detect the palm weevil using faster R-CNNs. In Figure 1, we illustrate some of the steps involved in detecting the palm weevil, such as generating a dataset, preparing the dataset for training, training the dataset using faster R-CNN and finally testing the trained model.

Deep learning is a state-of-the-art approach to classifying and identifying objects. Object detection automatically identifies the selected part of an image with the RPW. In the proposed method, faster R-CNN is used to detect the RPW and classify the species. The individual parts of an image are identified as enclosed in a selectable bounding box, and a class label is assigned to the RPW part of the image.

**Figure 2:**
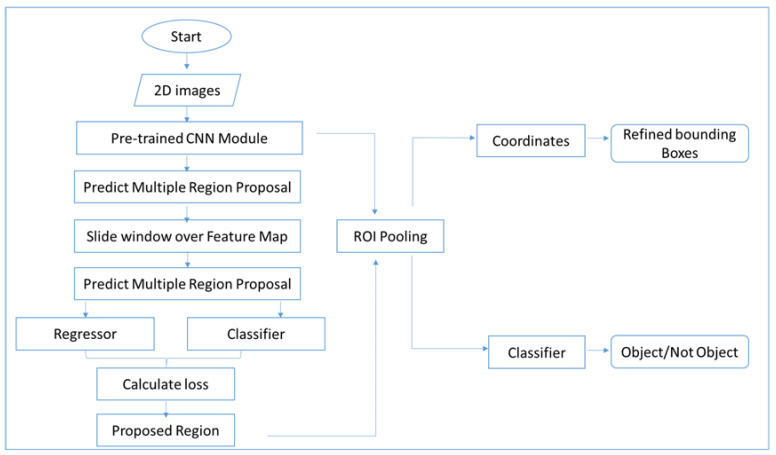
Block diagram of the proposed real-time system.

The faster R-CNN architecture consists of three major components, namely a convolutional layer (CNN), a region proposal network (RPN) and a class/bounding box detection mechanism. The CNN layer helps to discover the features of an image from data. The RPN works as a sliding window over the feature maps that have been extracted by the CNN. The last module, faster R-CNN, helps detect the bounding box of an object and the object itself. The whole mechanism of the architecture is explained in Figure 2. Two-dimensional images are passed to the CNN module, which detects multiple regions by using the sliding window of the CNN module to obtain a feature map (feature size: 60, 40, 512). In order to extract the feature vector, we applied the ROI region proposal to each region proposal. The output from the ROI pooling layer had a size of (N, 7, 7, 512), where N represented the number of proposals. The ROI layer helped in finding the exact coordinates/location of an object as well as categorizing the object as RPW or not.

### 3.1. Dataset Preparation

**Figure 3:**
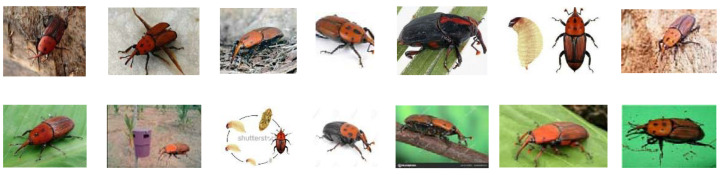
RPW dataset for the developed model.

For any computer-vision application, one of the most important tasks is generation of datasets for analysis. In order to perform this work, we downloaded 300 images of the palm weevil from the Google image search engine. Due to a non-availability of the dataset for the proposed model, the dataset was too small for the robust model to be trained. For this reason, data augmentation techniques were applied to the dataset. As shown in Table 1, there were different data augmentation techniques. Rotations were performed at various angles (−90°, −60°, −45°, −30°, 30°, 45°, 60°, 90°). Skewness and flip were also applied in all four directions. In addition, shear was applied at 10 and 20 degrees. Table 2 shows the statistics of the dataset before the augmentation and after the augmentation. The dataset was split into subfolders for training and testing. In the dataset, 80% of the data was used for training, whereas the remaining 20% was used for testing. Afterward, all of these images were converted into JPGs by using the JPG extension. XML files for each object were created by using labeling software to generate the X, Y coordinates of each object as shown in Figure 3, which illustrates the dataset used in this study.

### 3.2. Proposed Architecture

**Figure 4:**
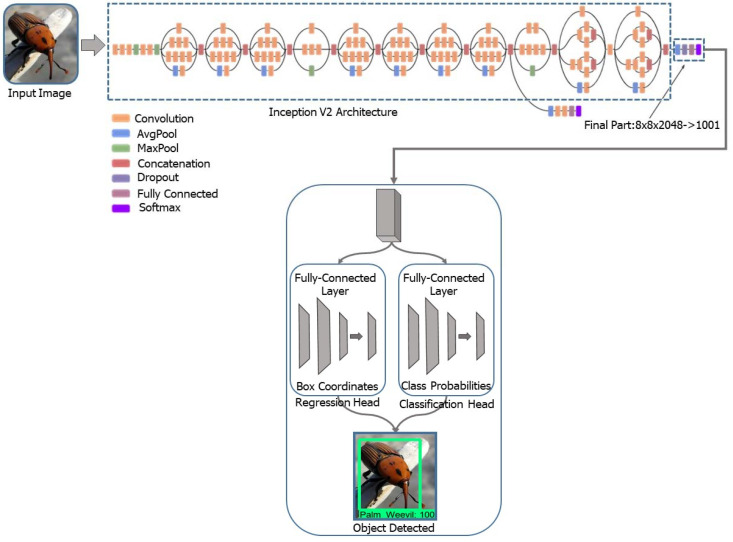
The overall architecture of the proposed model.

Once the dataset was ready, the next step as training. For training purposes, we used faster R-CNN, which can be easily deployed on embedded devices. We review the faster R-CNN detection framework briefly in this section. Faster R-CNN was first proposed for process object detection [12], in which an input image is given and the goal is to output a set of detection bounding boxes, each labeled with an object-class label. The complete pipeline consists of two stages: proposal generation and classification. First, an input image is processed by using 2D ConvNet to generate a 2D feature map. Another 2D ConvNet (referred to as the Region Proposal Network) is used to generate a sparse set of class-agnostic region proposals by classifying a set of variable-scale linking boxes centered at each pixel location of a feature map. The limits of the proposals are also adjusted with respect to linking boxes by regression. Second, for each region proposal, the features within the region are first aggregated into a feature map of constant size (i.e., RoI pooling [1]). Using the pooling feature, a DNN classifier then computes the probabilities of object classes and simultaneously regresses the detection limits for each object class. Figure 4 shows the entire pipeline. The framework is traditionally trained by alternating phase-one and phase-two training. Faster R-CNN naturally extends the temporal localization [13,14,15]. The aim of object detection is to detect 2D spatial regions, while in temporal procedure localization, the goal is to detect 1D temporal segments, each representing a start and end time. Thus, the temporal procedure localizes the 1D counterpart of object detection. A typical faster R-CNN pipeline for temporal procedure localization is as shown in Figure 4. Similar to object detection, it consists of two stages. First, given the sequence of frames, we extract a 1D feature map, usually via a 2D or 3D ConvNet. The feature map is then passed to 1D ConvNet 1 (referred to as the Segment Proposal Network) to classify a group of variable-size link segments at each temporal location and regress their boundaries. This returns a sparse set of class-agnostic segment proposals. Second, for each segment proposal, one computes the class probabilities and reviews the class-segment bounds further by first applying a 1D RoI pooling layer (termed “SoI pooling”) followed by a DNN classifier.

## 4. Results and Discussion

**Figure 5:**
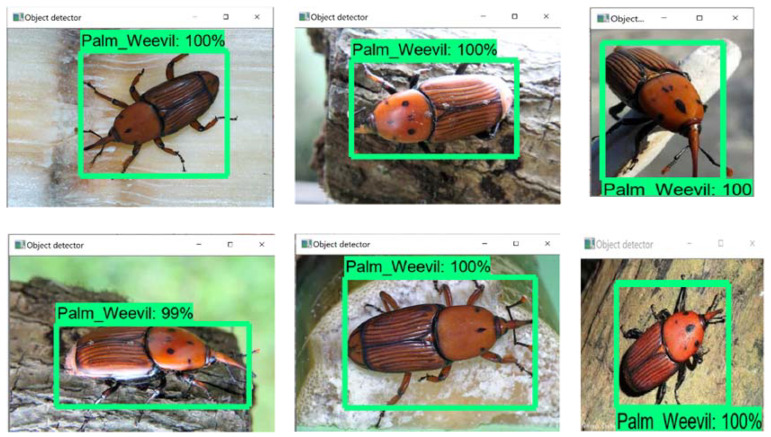
RPW results of the proposed model.

The use of deep learning in developing a model to accurately detect pests using imaginary localizations and measurements was proposed. This study used two classes, objects (palm weevil) and not-objects (backgrounds or other insects). As can be seen from Figure 5, the results obtained through the use of the developed model to determine the exact location of the RPW pest clearly showed an ability to distinguish the RPW pest from other insects. It is a well-known fact that RPW infestations cause significant changes in the trunk size of a palm tree compared to that of a non-infested one. Accordingly, the developed model can be used to detect the presence of the RPW before it enters the palm trunk. The performance of the developed model produced 100% results in terms of detection and classification.

Several runs were made to obtain the results for identifying the RPW. In this study, the proposed system network was evaluated by comparing different learning rates and by using different numbers of convolutional layers and different activation functions. We established that the proposed system network model is novel if one compares the results with those of other state-of- the-art models.

The proposed model was implemented in Python using TensorFlow along with Google’s Keras deep-learning framework. A Core i5 CPU evaluated the model’s training process by using an NVIDIA GeForce 1070, 8 GB GPU and 24 GB of RAM. The model was trained over a period of 200 epochs.

For training the proposed faster R-CNN method, a binary label was assigned to each anchor. A positive label was assigned to an anchor that had an IoU (intersection over union) greater than 0.7 for any ground-truth box, while a negative label was assigned to an anchor that had less than 0.3 for all ground-truth boxes. The loss function of the proposed model for an image was defined as:

(1) $$L\left(\left\{P_{n}\right\},\left\{t_{n}\right\}\right)=\frac{1}{N_{clc}}\sum_{n}L_{clc}\left(p_{n},p_{n}^{*}\right)+\frac{1}{N_{reg}}\sum_{n}p_{n}^{*}L_{reg}\left(t_{n},t_{n}^{*}\right)$$

Here:

*N* = Index of an anchor

*P_n_* = Predicted probability of an anchor

$p_{n}^{*}$ = Ground-truth label (one in a positive case and zero in a negative case)

*t_n_* = Four coordinates of a predicted bounding box

*L_clc_* = Classification loss

*L_reg_* = Regression loss

ʎ = Balance weight

$t_{n}^{*}$ = Ground-truth box associated with a positive anchor

*N_clc_* = Classification normalization

*N_reg_* = Regression normalization

**Figure 6:**
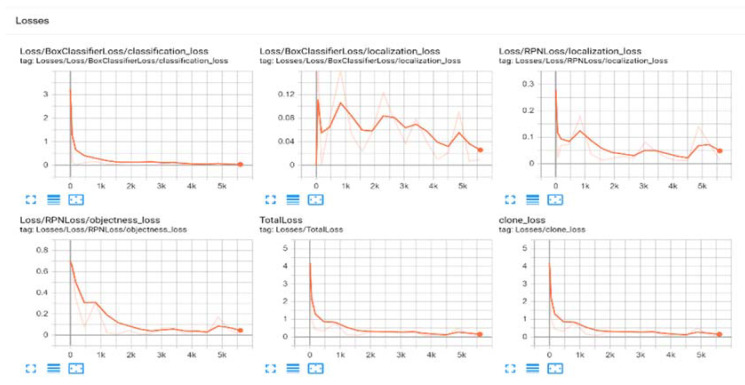
Graphical results of the proposed model.

Figure 6 shows the multi-loss graphs of the proposed model. It includes classification loss, localization loss, objectness loss, total loss and clone loss. The classification loss *L_clc_* is the log loss over the two classes (object and not object). The regression loss *L_reg_* is concerned with the parameterization of the four coordinates (x, y, width and height), is activated for only a positive anchor ($p_{n}^{*}\;$= 1) and is deactivated in other cases. The objectness loss shows the positions of oriented factors and the horizontal labels. The clone loss is concerned with lessening the within-class variance in features.

Table 3 shows the comparative analysis of the proposed work with state-of-the art algorithms [20]. For the detection of the RPW, the SVM, MLP, AdaBoost and Random Forest algorithms each showed a 93.08% accuracy, whereas the Naïve Bayes algorithm showed an 82.58% accuracy. Using the proposed model (faster R-CNN), 99% of the RPW cases were classified and located accurately.

## 5. Conclusions

It should be noted that in the past few decades, the spread of the RPW, a detrimental pest infesting palm trees, has increased drastically. With the objective of reducing palm tree losses and the manipulation of this pest, this paper presented the development of a model that can detect an infestation without having to sacrifice trees. Therefore, the primary objective of the paper was to develop a model that can detect and classify the red palm weevil pest and differentiate it from different types of insect pests. According to the developed model, RPW infestations can be accurately detected. In order to demonstrate the effectiveness of the developed model in different areas, a localization and classification approach was applied [21]. For the purposes of cross-validation using a real dataset of RPWs, the model was run by using different sizes of datasets. In the study, the ability of the developed classification model to detect the RPW was compared to that of other models. In terms of detection accuracy, the overall performance results of the classification model came out as 99%. It was shown in the literature that there are other models of deep learning that are more robust than faster R-CNN in the detection of real-time objects. In the future, YOLO can also be utilized for the detection of RPWs [22,23]. For the survival of the trees, it is highly important to use an effective method for the early detection of infestations by the RPW in various types of trees.

## Figures and Tables

**Table 1:** Different techniques of data augmentation.

S. No.	Data Augmentation Technique	Parameter(s)
1	Rotation	−90° −60° −45° −30° 30° 45° 60° 90°
2	Skewness	Right Left Forward Backward
3	Flip	Bottom Top Left Right
4	Shear	Along *X*-axis at 10°, 20° Along *Y*-axis at 10°, 20°

**Table 2:** Statistics of red palm weevil dataset.

Dataset	No. of Images before Augmentation	Parameters of Augmentation Techniques	No. of Images after Augmentation
Red palm weevil	300	20	6000

**Table 3:** Comparative analysis of the proposed model.

S. No.	Algorithm	Accuracy
1	SVM	93.08%
2	Naive Bayes	82.58%
3	Random Forest	93.08%
4	MLP	93.08%
5	AdaBoost	93.08%
6	Faster R-CNN	99%

## Data Availability

Not applicable.
